# Rituximab in patients with membranous nephropathy and kidney insufficiency

**DOI:** 10.3389/fphar.2022.1002117

**Published:** 2022-10-10

**Authors:** Yanhong Guo, Liuwei Wang, Yulin Wang, Xiaodan Li, Zihan Zhai, Lu Yu, Yan Liang, Peipei Liu, Lin Tang

**Affiliations:** ^1^ Department of Nephropathy, the First Affiliated Hospital of Zhengzhou University, Zhengzhou, China; ^2^ Clinical Systems Biology Laboratories, the First Affiliated Hospital of Zhengzhou University, Zhengzhou, China

**Keywords:** rituximab, membranous nephropathy, kidney insufficiency, anti- PLA2R antibody, CD19 B cells +

## Abstract

**Introduction:** Patients with membranous nephropathy and kidney insufficiency have an extremely high risk of progression to end-stage renal disease. Whether rituximab can effectively treat membranous nephropathy patients with renal dysfunction remains unknown at present. The aim of our study was to evaluate the effectiveness and safety of rituximab (RTX) in membranous nephropathy with kidney insufficiency.

**Methods:** We retrospectively analyzed the clinical data of 35 membranous nephropathy patients with kidney insufficiency administered in the First Affiliated Hospital of Zhengzhou University between January 2020 and December 2021. Patients were followed every 1–3 months for a total of 6 months. Clinical data were collected including anti-phospholipase A2 receptor antibody (anti-PLA2R antibody) quantification, 24-h urinary protein, serum albumin, and serum creatinine. The percentage of patients who achieved clinical remission was measured.

**Results:** There were 7 (20%) patients who achieved complete or partial response at 6 months after RTX treatment. After 6 months of treatment, patients were clinically improved as evidenced by significant improvements in anti- PLA2R antibody titer [7.70 (5.72, 16.72) vs. 59.20 (17.70, 187.50) RU/ml, *p* < 0.001], 24-h urine protein [7.04 (4.43, 8.90) vs. 10.15 (4.83, 13.57) g/d, *p* < 0.001], serum albumin [30.55 (24.97, 33.27) vs. 21.40 (16.75, 25.00)g/L, *p* < 0.001], serum creatinine [99.50 (75.25, 140.25) vs. 152.00 (134.50, 232.50) µmol/L, *p* = 0.022], and estimated glomerular filtration rate (eGFR) [78.29 (50.15, 101.55) vs. 41.12 (26.53, 51.41) ml/min/1.73 m^2^, *p* = 0.045]. There were no significantly differences between responders and nonresponders in the baseline levels of anti-PLA2R antibodies, proteinuria, serum albumin, and renal function. After the RTX treatment, anti-PLA2R antibodies turned negative in all responders, but the antibody level persisted maintained positive in all but 5 nonresponders. The patients who achieved response maintained a stable kidney function during the study period, with eGFR 29.03 (28.76, 35.07) ml/min/1.73 m^2^ before rituximab treatment and 62.73 (62.34, 63.13) ml/min/1.73 m^2^ at the end of follow-up (*p* = 0.053).

**Conclusion:** RTX therapy might be an alternative treatment in reducing proteinuria and maintaining stable renal function among membranous nephropathy patients even with kidney insufficiency.

## 1 Introduction

Membranous nephropathy is one of the most common causes of nephrotic syndrome in adults without diabetes and the most common pathological type of nephrotic syndrome (du Buf-Vereijken et al., 2005), accounting for approximately one-third of biopsied nephrotic syndrome cases (Francis et al., 2016). The prognosis of membranous nephropathy varies, and approximately 30–40% of patients progress toward end stage renal disease within 5–15 years, especially for the patients with persistent proteinuria or kidney insufficiency (Lai et al., 2015; Trujillo et al., 2020). The latest Kidney disease Improving Global Outcomes (KDIGO) guidelines published in 2021 recommend that glucocorticoids in combination with cyclophosphamide should be the first choice for membranous nephropathy patients at very high-risk of progression to end stage renal disease (Rovin et al., 2021). However, this regimen is associated with severe side effects including infection and reproductive toxicity (Ghobadi et al., 2017; Liu et al., 2019).

Rituximab (RTX) is a human/murine chimeric monoclonal antibody targeting CD20 molecule on the surface of pre-B cells and mature B lymphocytes (Pescovitz, 2006). RTX could induce the apoptosis of B-cell and reduce autoantibody production including anti-phospholipase A2 receptor antibody (anti-PLA2R antibody) (Pescovitz, 2006; van de Logt et al., 2018). The 2021 KDIGO guidelines also recommend the use of RTX as the first-line therapy for patients with membranous nephropathy at moderate to high-risk of progressive loss of kidney function (Rovin et al., 2021). However, whether RTX can be safe and effective in inducing remission of nephrotic syndrome, preserving renal function, and delaying the progression of chronic kidney disease to end-stage renal disease in patients with membranous nephropathy and kidney insufficiency remains unclear. This was the first study to investigate the effectiveness and safety of RTX in the management of Chinese patients with membranous nephropathy and kidney insufficiency.

## 2 Methods

### 2.1 Study design and patients

A total of 35 patients administered in the First Affiliated Hospital of Zhengzhou University between January 2020 and December 2021 with the following criteria were included in this study: 1) All patients were diagnosed of membranous nephropathy by kidney biopsy and were aged ≥18 years at baseline; 2) There were no secondary causes identified for membranous nephropathy. We excluded common causes of secondary membranous nephropathy including infections, autoimmune diseases, malignancy, sarcoidosis, or heavy metal poisoning by examination including anti-hepatitis B virus/hepatitis C virus, antinuclear antibody, anti-double-stranded DNA, extractable nuclear antigen antibody, tumor markers, chest computed tomography, urinary mercury and so on. 3) Kidney insufficiency was defined as a serum creatinine level of 130 μmol/L or greater; 4) Patients were treated with RTX for membranous nephropathy.

To assess treatment response, complete remission of nephrotic syndrome was defined as 24-h urine protein <0.3 g/day. Partial response was defined as proteinuria >0.3 g/d but <3.5 g/d or a reduction of proteinuria by ≥ 50% of the initial value and <3.5 g/d (Rovin et al., 2021). Patients who did not meet the above definition at 6 months after RTX treatment were defined as nonresponders.

For most patients, the treatment regimen consisted of once weekly RTX at a dose of 375 mg/m^2^ for a total of 4 sessions or 1g RTX 2 weeks apart for a total of 2 sessions. Treatment was repeated if needed to achieve the complete depletion of anti-PLA2R antibody. There is also a small proportion of membranous nephropathy patients, who are given a dose of RTX less than the two regimens mentioned above, depending on the immune status and whether the anti-PLA2R antibody turned negative or not. The number of RTX injections, doses per injection, and dosing interval were left to the discretion of the treating physicians. The research was approved by the ethics committee of the First Affiliated Hospital of Zhengzhou University. Informed consents were obtained from those patients.

### 2.2 Clinical data collection

Patients’ clinical and laboratory parameters were collected before RTX administration, and were repeated on every visit in month 3 and 6 after RTX administration. A follow-up period greater than 6 months was required for all patients. Follow up ended when patients received other immunosuppressive agents or received dialysis.

Prior to RTX administration, general clinical parameters including gender, age, blood pressure, previous treatment regimens, and current combination therapies were collected. At subsequent 3 months intervals after RTX administration, laboratory variables related with nephrotic syndrome and renal function were collected.

Anti-PLA2R antibodies were detected using a commercial enzyme-linked immunosorbent assay (ELISA) kit (EUROIMMUN AG, Lübeck, Germany). Blood urea nitrogen (BUN), and serum albumin were measured by colorimetry using Cobas c 701 (Roche, Basel, Switzerland). Serum creatinine was measured by Enzymatic methods using Cobas c 701 (Roche, Basel, Switzerland). The amount of proteinuria was evaluated by 24-h urine protein. The estimated glomerular filtration rate (eGFR) was calculated using the Chronic Kidney disease Epidemiology Collaboration (CKD-EPI) formula.

### 2.3 Statistical analysis

Patient characteristics were described by mean ± standard deviation, medians, interquartile ranges, and percentages according to data type. If the data are in accordance with normal distribution, independent sample *t*-test is used for comparison between the two groups. One-way ANOVA was used for comparison among multiple groups, and Bonferroni correction was used for pairwise comparison between groups if the data met normal distribution and homogeneity of variance. If the measurement data do not conform to the normal distribution, Mann-Whitney *U* test is used for comparison between the two groups. For data that were not normally distributed or had unequal variance, Kruskal - Wallis H tests were used for multiple comparisons, with Bonferroni correction for pairwise comparisons between groups. Categorical variables were expressed by the number of cases and percentage (%), and comparisons between groups were performed by x^2^ test or Fisher’s exact test. All of these data were analyzed using the SPSS 23.0 software package and *p*-values were calculated as two-sided. Statistical significance was set at *p* < 0.05.

## 3 Results

### 3.1 Baseline characteristics at rituximab Infusion

From January 2020 and December 2021, 35 consecutive membranous nephropathy patients with renal insufficiency were enrolled in the present study. Their demographic and clinical data at RTX infusion were shown in Table 1. There were 5 female and 30 male patients, with an average age of 54.44 ± 12.77 years old. They had experienced a membranous nephropathy history of 35.50 (15.00, 53.55) months since their percutaneous renal biopsy operation. Among them, 34 patients were diagnosed as PLA2R associated with membranous nephropathy according to glomerular PLA2R immunohistochemistry staining or the examination of serum anti-PLA2R antibodies. Three patients received RTX therapy as the initial treatment, and 32 patients had been administered at least one course of immunosuppressive therapy, including corticosteroid plus cyclophosphamide in 12 patients, cyclosporine in 12 patients, tacrolimus in 27 patients, and mycophenolate mofetil in 4 patients.

**TABLE 1:** Baseline characteristics of MN patients with renal insufficiency.

Variables	All patients (n = 35)
Demographics	
Gender (male/female)	30/5
Age (year)	54.44 ± 12.77
BP (mmHg)	
Systolic	130.87 ± 10.50
Diastolic	79.33 ± 8.65
Creatinine (µmol/L)	171.00 (136.00, 217.00)
eGFR (ml/min/1.73m^2^)	40.99 (26.72, 50.50)
eGFR 30–60	24
eGFR 15–29	9
eGFR<15	2
24 h urine protein (g/d)	10.33 (7.13, 13.69)
Albumin (g/L)	21.51 ± 5.99
Median time since biopsy-proven diagnosis (month)	35.50 (15.00, 53.25)
anti- PLA2R-Ab titer (RU/mL)	47.40 (17.65, 187.50)
Diuretics (N, %)	7 (20.00%)
ACE inhibitor or ARB (N, %)	1 (2.85%)
Statins (N, %)	15 (42.85%)
Previous therapies	
Prednisone + cyclophosphamide, n	12
Prednisone + cyclosporine, n	12
Tacrolimus, n	27
Mycophenolate mofetil, n	4
Number (%) of patients with	
One round of immunomeds	19 (54.28%)
Two rounds of immunomeds	10 (28.57%)
Three rounds of immunomeds	3 (8.57%)

Data presented as median (first-third interquartile range) or mean ± SD, or number (percentage). BP, blood pressure; eGFR, estimated glomerular filtration rate; anti- PLA2R-Ab, anti-phospholipase A2 receptor antibody; ACE inhibitor, angiotensin-converting enzyme inhibitors; ARB, angiotensin II receptor blocker.

By the time of enrollment, serum anti-PLA2R antibody was measured with a median titer of 47.40 (17.65, 187.50) RU/mL. Among all patients, the level of proteinuria was 10.33 (7.13, 13.69) g/24 h, and the mean serum albumin level was 21.51 ± 5.99 g/L. The median serum creatinine level was 171.00 μmol/L (IQR, 136.00–217.00), and eGFR was 40.99 ml/min/1.73m^2^ (IQR, 26.72–50.50). Among them, 24 patients have an eGFR ranging from 30–60 ml/min/1.73m^2^, and 9 patients have an eGFR ranging from 15–29 ml/min/1.73m^2^, and the remaining 2 patients have an eGFR less than 15 ml/min/1.73m^2^ (Table 1).

### 3.2 Clinical and immunological outcomes

All the patients were followed up every 1–3 months for 6 months. There were 7/35 (20%) patients who achieved complete or partial response at 6 months.

Median CD19^+^ B cell count was 179/mm^3^ (IQR, 85.00–272.50) at baseline. Circulating CD19^+^ B cells at 3 months were significantly reduced compared with the baseline levels (*p* < 0.001). At 6 months, the number of CD19^+^ B cells had recovered to some extent, and there was no significant difference between the baseline level and that 6 months after RTX intervention.

34 patients in this study were PLA2R associated membranous nephropathy, and anti- PLA2R antibody was detectable in 34 (97.14%) patients at baseline. At 3 months, the percentage of anti-PLA2R antibody positive patients (71.42 versus 97.14%; *p* = 0.003) and anti-PLA2R antibody titers (8.60; IQR, 0.00, 42.50 vs. 59.20; IQR, 17.70, 187.50 RU/ml; *p* = 0.001) were lower compared with the baseline levels (Table 2). No significantly differences in the percentage of anti-PLA2R-antibody positive patients and anti-PLA2R-antibody titer were observed between 3 and 6 months. Complete immunologic remission (full anti-PLA2R-antibody depletion) was observed in 9 of 35 (26.47%) and 10 of 35 (29.41%) patients at 3 and 6 months.

**TABLE 2:** Efficacy outcome variables.

Variables	Baseline	3 months	6 months
Remission, complete and partial (N, %)	NA	NA	7 (20%)
24 h urine protein (g/d)	10.15 (4.83, 13.57)	6.02 (4.83, 11.24)^a^	7.04 (4.43, 8.90)^b^
Albumin (g/L)	21.40 (16.75, 25.00)	24.80 (22.05, 29.90)^a^	30.55 (24.97, 33.27)^b^
Serum creatinine (µmol/L)	152.00 (134.50, 232.50)	136.00 (126.00, 214.50)	99.50 (75.25, 140.250)^b^
eGFR (ml/min/1.73 m^2^)	41.12 (26.53, 51.41)	47.85 (30.54, 53.86)	78.29 (50.15, 101.55)^b^
anti-PLA2R-Ab–positive patients (N, %)	34 (97.14%)	25 (71.42%)^a^	24 (68.57%)^b^
anti-PLA2R-Ab–depleted patients (N, %)	NA	9/34 (26.47%)	10/34 (29.41%)
anti- PLA2R-Ab titer (RU/mL)	59.20 (17.70, 187.50)	8.60 (0.00, 42.50)^a^	7.70 (5.72, 16.72)^b^
CD19, per mm	179.00 (85.00, 272.50)	1.00 (0.00, 3.00)^a^	25.50 (3.75, 57.75)
CD4, per mm	893.00 (561.00, 1,095.00)	718.00 (460.50, 908.50)	816.00 (417.00, 1,031.25)
Post hoc composite end point at 6 months	NA	NA	16 (45.71%)

Data presented as number (percentage) or median (first-third interquartile range).^a or b^ Stands for *p* < 0.05 vs. baseline. BP, blood pressure; eGFR, estimated glomerular filtration rate; anti- PLA2R-Ab, anti-phospholipase A2 receptor antibody; CD, cluster of differentiation; Complete and partial remissions were defined according to 2021 KDIGO criteria on the basis of proteinuria; composite end point was defined as reduction of proteinuria >50% and increase of serum albumin >30%.

**FIGURE 1:**
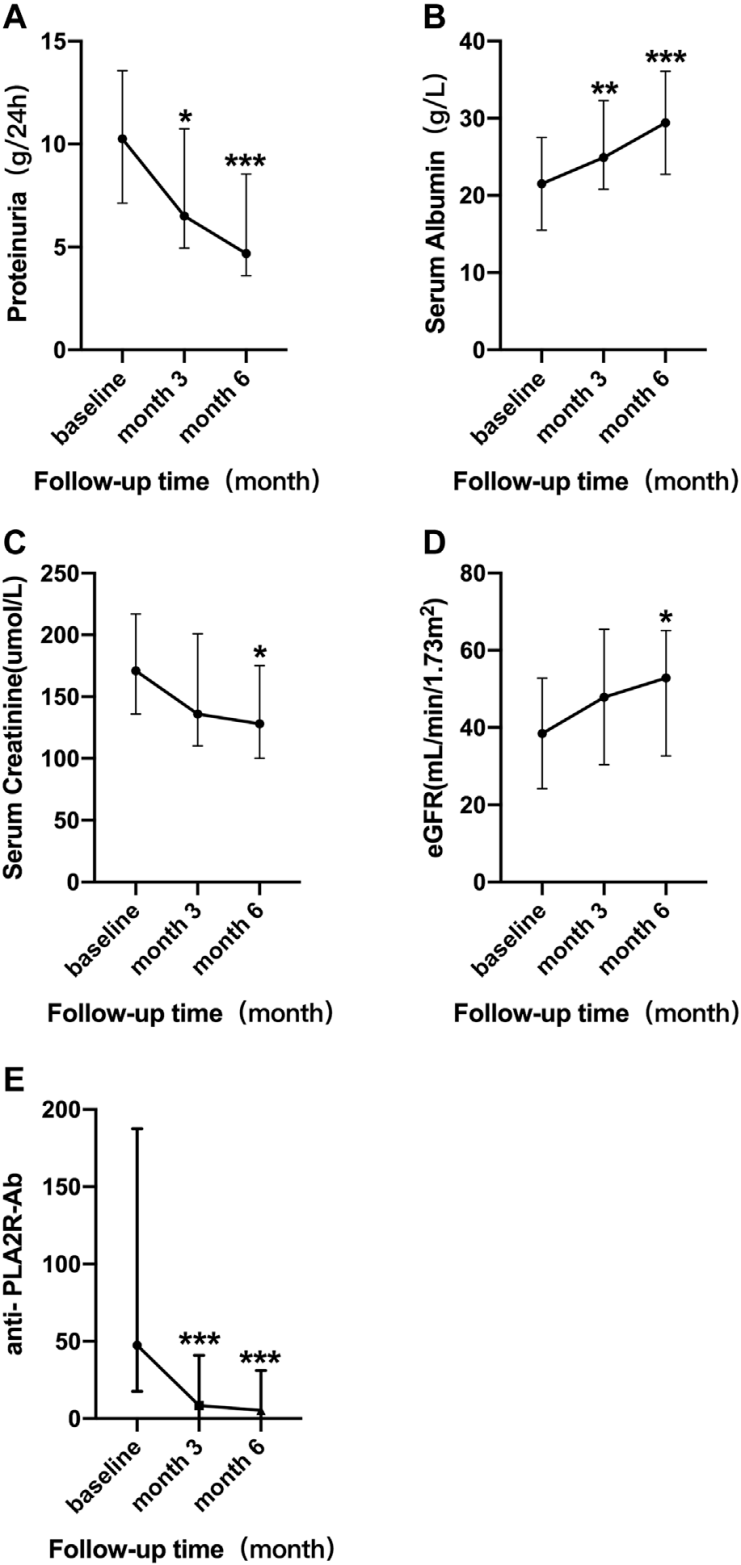
Serial levels of 24 h urinary protein **(A)**, serum albumin **(B)**, serum creatinine **(C)**, eGFR **(D)**, and anti-PLA2R antibody **(E)** after the rituximab treatment in all patients who had been followed up for 6 months. The bars show median with interquartile range for each variable. *p < 0.05 vs. baseline; **p < 0.01 vs. baseline; ***p < 0.001 vs. baseline.

In addition to anti- PLA2R antibody, serial monitoring of the membranous nephropathy patients who had been followed-up for 6 months are shown in Figure 1 for proteinuria, serum albumin, creatinine, and eGFR after RTX treatment. The results showed that after 3 months of RTX treatment, compared with the baseline levels, the level of 24-h urinary protein decreased significantly [ 6.02 (4.83, 11.24) vs. 10.15 (4.83, 13.57) g/d, *p* = 0.035] and serum albumin increased significantly [24.80 (22.05, 29.90) vs. 21.40 (16.75, 25.00) g/L, *p* < 0.001]. Unlike the above indicators, there was no significant improvement in renal function after 3 months of RTX treatment including serum creatinine [136.00 (126.00, 214.50) vs. 152.00 (134.50, 232.50) µmol/L, *p* = 0.115] and eGFR [47.85 (30.54, 53.86) vs. 41.12 (26.53, 51.41)) ml/min/1.73m^2^, *p* = 0.184]. After 6 months of treatment, patients were clinically improved as evidenced by significant improvements in anti- PLA2R antibody [7.70 (5.72, 16.72)vs. 59.20 (17.70, 187.50) RU/ml, *p* < 0.001], 24-h urine protein [7.04 (4.43, 8.90) vs. 10.15 (4.83, 13.57) g/d, *p* < 0.001], serum albumin [30.55 (24.97, 33.27) vs. 21.40 (16.75, 25.00) g/L, *p* < 0.001], serum creatinine [99.50 (75.25, 140.25) vs. 152.00 (134.50, 232.50) µmol/L, *p* = 0.022], and eGFR [78.29 (50.15, 101.55) vs. 41.12 (26.53, 51.41) ml/min/1.73m^2^, *p* = 0.045]. These results also suggest that in the treatment of membranous nephropathy with RTX the improvement of renal function lags behind the remission of proteinuria (Table 2).

### 3.3 Comparison between responders and nonresponders at rituximab initiation and the end of follow-up after rituximab treatment

There were no significant differences in the baseline levels of proteinuria, serum albumin, and renal function between responders and nonresponders (Table 3). The total dose of RTX was comparable between the two groups [2.00 (1.20, 2.00) vs. 2.00 (2.00, 2.00)g, *p* = 0.294]. In the 34 PLA2R associated patients, the baseline antibody level was lower in the 7 responders at RTX initiation. However, the difference is not statistically significant [5.90 (5.70, 34.20) vs. 71.80 (45.40, 257.90) RU/ml, *p* = 0.531]. After the RTX treatment, anti-PLA2R antibodies turned negative in all responders, but the antibody level maintained positive in all but 5 non-responders.

**TABLE 3:** Comparison between responders and nonresponders at rituximab initiation.

Variables	Responders (*N* = 7)	Nonresponders (*N* = 28)	P-Valve
Demographics			
Gender (male/female)	6/1	24/3	0.855
Age (year)	53.00 (42.50, 59.00)	55.50 (50.25, 68.25)	0.708
BP (mmHg)			
Systolic	132.43 ±7.99	130.46 ± 11.18	0.667
Diastolic	81.71 ± 6.34	78.69 ± 9.17	0.421
Serum creatinine (µmol/L)	171.00 (161.50, 209.50)	140.00 (133.00, 217.00)	0.672
eGFR (ml/min/1.73m^2^)	29.03 (28.76, 35.07)	45.09 (26.13, 53.75)	0.835
24 h urinary protein (g/d)	7.92 (4.19, 14.00)	11.21 (5.22, 13.35)	0.624
Albumin (g/L)	23.30 (20.20, 29.25)	19.90 (16.40, 23.80)	0.183
CD19, per mm	241.00 (165.50, 249.50)	166.50 (80.00, 278.00)	0.800
CD4, per mm	1,059.00 (945.00, 1,315.00)	812.50 (522.50, 962.00)	0.317
Median time since biopsy-proven diagnosis (month)	30.00 (15.50, 39.00)	35.50 (16.00, 54.00)	0.559
anti- PLA2R-Ab titer (RU/mL)	5.90 (5.70, 34.20)	71.80 (45.40, 257.90)	0.531
Diuretics (N, %)	0 (0.0%)	7 (25.0%)	0.176
ACE inhibitor or ARB (N, %)	0 (0.0%)	2 (7.1%)	0.635
Statins (N, %)	2 (28.6%)	13 (46.4%)	0.340
Number (%) of patients with			
Zero round of immunomeds	1 (14.3%)	2 (7.1%)	0.778
One round of immunomeds	4 (57.1%)	15 (53.6%)	
Two rounds of immunomeds	2 (28.6%)	8 (28.6%)	
Three rounds of immunomeds	0 (0.0%)	3 (10.7%)	
rituximab dose (g)	2.00 (1.20, 2.00)	2.00 (2.00, 2.00)	0.294

Data presented as median (first-third interquartile range) or mean ± SD, or number (percentage). *Stands for *p* < 0.05. BP, blood pressure; eGFR, estimated glomerular filtration rate; CD, cluster of differentiation; anti- PLA2R-Ab, anti-phospholipase A2 receptor antibody; ACE inhibitor, angiotensin-converting enzyme inhibitors; ARB, angiotensin II receptor blocker.

The patients who achieved response maintained a stable kidney function during the study period, with eGFR 29.03 (28.76, 35.07) ml/min/1.73 m^2^ before RTX treatment and 62.73 (62.34, 63.13) ml/min/1.73 m^2^ at the end of follow-up (*p* = 0.053). The nonresponders showed no significant difference in kidney function with serum creatinine changing from 140.00 (133.00, 217.00) to 113.50 (85.00, 128.00) µmol/L (*p* = 0.051) and eGFR changing from 45.09 (26.13, 53.75) on enrollment to 57.74 (48.18, 100.37) ml/min/1.73 m^2^ (*p* = 0.063) at the end of follow-up (Table 3 and Table 4). One patient entered into dialysis at month 6 after RTX treatment.

**TABLE 4:** Clinical characteristics of responders and nonresponders by the end of follow-up after rituximab treatment.

Variables	Responders (*N* = 7)	Nonresponders (*N* = 28)	P-Valve
Proteinuria by the end of follow-up (g/24 h)	1.22 (0.37, 2.08)	5.34 (4.06, 8.54)	<0.001*
Albumin by the end of follow-up (g/L)	35.55 (32.30, 38.80)	29.65 (23.60, 32.00)	<0.001*
Creatinine by the end of follow-up (µmol/L)	117.50 (106.00, 129.00)	113.50 (85.00, 128.00)	0.929
eGFR by the end of follow-up (mL/min/1.73 m^2^)	62.73 (62.34, 63.13)	57.74 (48.18, 100.37)	0.755
Anti-PLA2R-Ab titer (RU/mL)	0.00 (0.00, 0.00)	9.80 (7.00, 12.00)	0.031*
Change of eGFR (mL/min/1.73 m^2^)	22.01 (7.68, 33.85)	5.01 (−8.68, 24.86)	0.281

Data presented as median (first-third interquartile range). *Stands for *p* < 0.05. BP, blood pressure; eGFR, estimated glomerular filtration rate; anti- PLA2R-Ab, anti-phospholipase A2 receptor antibody.

### 3.4 Safety analysis of rituximab used in membranous nephropathy patients with kidney insufficiency

RTX was well tolerated by all 35 patients. No serious adverse events were observed during the infusion in all patients. Only 1 patient experienced an anaphylactic reaction during infusion, manifested as a skin rash, and the symptoms completely disappeared after antiallergic treatment.

## 4 Discussion

Cyclical cyclophosphamide plus glucocoticoids is often used in membranous nephropathy patients with kidney insufficiency (Ramachandran et al., 2016; Ramachandran et al., 2022), and is also recommended as the first-line agents by the 2021 KDIGO guidelines. However, cyclophosphamide is not widely used in clinical practice among young patients with fertility requirements or patients with poor immunity because of reproductive toxicity and strong immunosuppression. At present, RTX is widely used in the treatment of membranous nephropathy, and it is also recommended by the 2021 KDIGO guidelines for the treatment of membranous nephropathy at moderate to high-risk of progressive loss of kidney function (Rovin et al., 2021). However, for patients with membranous nephropathy and kidney insufficiency, whether RTX can effectively decrease proteinuria, preserve renal function, and delay or even reverse the progress of renal failure is still controversial (Wang et al., 2018; Hanset et al., 2020; Ramachandran et al., 2022).

Our study showed that RTX treatment was effective in 20% of patients with membranous nephropathy and kidney insufficiency at 6 months. Although the complete or partial remission rate in our study is not as effective as previously reported response rates of approximately 60% (Teisseyre et al., 2021; Ou et al., 2022), RTX therapy appears to remain a treatment option for patients with membranous nephropathy with kidney insufficiency.

There are several reasons for the differences in remission rates between our study and previous trials. Firstly, 91.42% of the patients enrolled in this study had previously received 1-3 rounds of immunosuppressive regimens and 21 patients never had remission of nephrotic syndrome, so these patients may be resistant to another immunosuppressive drug including RTX. Secondly, all patients in this study had renal dysfunction, and the median serum creatinine level was 171 μmol/L, suggesting the possible existence of renal tubulointerstitial damage and impaired renal function. Previous studies have shown that patients with tubulointerstitial disease and impaired renal function respond worse than patients with normal renal function (Peponis et al., 2010). In this study, RTX still induced a better treatment response in a subset of patients with acute or chronic renal impairment. Thirdly, the dose of RTX may be insufficient for some patients in this study. The optimal dose of RTX in the treatment of membranous nephropathy remains controversial (Moroni et al., 2017; Bagchi et al., 2018; Fenoglio et al., 2021). Cravedi *et al.* have found that low-dose RTX can reduce cost, and some small sample studies confirmed that low-dose RTX can effectively induce the remission of membranous nephropathy (Cravedi et al., 2007; Fenoglio et al., 2021). However, most studies demonstrated that compared with low-dose RTX, high-dose RTX can induce remission of membranous nephropathy more effectively (Seitz-Polski et al., 2019). The dose of RTX varied in our study, ranging from 4 weekly 375 mg/m^2^ RTX infusion or 2 RTX infusions of 1 g/d 2 weeks apart, but the total dose with RTX was mostly 2 g. However, there are still some patients due to economic status or immune status that lead to insufficient use of RTX, resulting in incomplete immunological remission. There are also some patients who decide the dose of RTX based on whether complete B-cell depletion is achieved, but CD20^+^ B cell burdens are generally lower in membranous nephropathy patients who have undergone prolonged immunosuppressive therapy in our study. Lower dose of RTX might be required to induce complete CD20^+^ B cell depletion. However, depletion of B lymphocytes alone may not be an effective means to determine the optimal dose of RTX, and it cannot achieve complete immunological remission (Cravedi et al., 2007; Moroni et al., 2017). Therefore, in order to exclude the poor outcomes caused by insufficient dosage of RTX, the standard dose of RTX when the immune status of patients allows may be necessary. Fourthly, the follow-up time of patients was shorter than previous studies which follow-up periods were typically as long as 12–24 months, or even longer (van den Brand et al., 2017; Fernández-Juárez et al., 2021). Some of these patients in our study had achieved immunological remission at the end of the 6-months follow-up, and although complete or partial remission of proteinuria had not been achieved, there is hope that clinical remission will be achieved in the future. Besides that, it has been confirmed that anti- PLA2R antibody titer at baseline was an independent predictor of immunological remission after 6 months of RTX treatment. Disappearance of anti-PLA2R antibody was less likely in patients treated with RTX for 6 months with an anti- PLA2R antibodies titer higher than 152 RU/ml (van de Logt et al., 2018). In this study, although no statistical difference was found due to the small sample size, the anti- PLA2R antibody level of nonresponders seemed to be higher than those of responders, which was also one of the reasons for the poor outcome at 6 months. In the nonresponders group, although not all patients achieved immunological remission after 6 months of treatment, anti-PLA2R antibodies were significantly decreased at 6 months after the treatment of RTX. We speculate that those patients still have the chance of achiving remission if RTX treatment is repeated in the subsequent follow up.

Except for the above factors, there are several factors including serum RTX levels, the existence of anti- RTX antibodies, Th17-mediated inflammation and so on that have been demonstrated to influence the efficacy of RTX in the treatment of membranous nephropathy (Teisseyre et al., 2022). Studies have confirmed that low RTX levels at month 3 correlate with poor B-cell depletion at months 3 and 6, with high anti-PLA2R antibody titer at months 3, 6 and 12, and with proteinuria at months 3, 6 and 12. Close monitoring of serum RTX levels can improve the efficacy of RTX. Serum RTX levels are related to drug dose, and proteinuria (Jacobs et al., 2017). While, a large inter-individual variability among patients even treated with the same schedule of RTX may also correlate with serum RTX levels. RTX recycling depends on endothelial cells via FcRn, and the efficacy of recycling seems different among membranous nephropathy patients due to the polymorphism of FcRn (Boyer-Suavet et al., 2019a). However, serum RTX levels cannot be tested in our hospital at present. In the future, we hope to carry out this examination to identify patients who are likely to be resistant or less effective to RTX, and improve the efficacy of the treatment. Anti- RTX antibodies appeared in 23% of patients which resulted in faster B cell reconstitution (Boyer-Suavet et al., 2019b). Therefore, in patients who have received RTX treatment, especially in patients who have relapsed, anti- RTX antibody should be detected. Poor outcomes with RTX in membranous nephropathy may also be associated with Th17-mediated inflammation which is associated with severe complications including thrombosis and relapses. However, RTX treatment did not impact Th17 cytokines (Cremoni et al., 2020). The detection of Th17 mediated inflammation at diagnosis is helpful for the choice of treatment options. Treatment inducing re-orientation of the immune system could be considered for those patients.

Our study demonstrated that after treatment with RTX anti-PLA2R antibody level was decreased significantly in patients with membranous nephropathy and kidney insufficiency. Compared with nonresponders, the responders had lower levels of anti-PLA2R antibodies at 6 months after the RTX infusion. These results suggest that the disappearance of anti-PLA2R antibodies after RTX treatment correlates with clinical remission, indicating that immunological remission may be an indicator of renal survival after treatment with RTX for patients with membranous nephropathy and renal dysfunction.

The lack of comparison with cyclophosphamide plus glucocoticoids is one of the limitations of this manuscript. Clinical studies about the efficacy of cyclophosphamide in the treatment of membranous nephropathy with kidney insufficiency showed in Table 5. The studies showed that cyclophosphamide can be useful in membranous nephropathy with kidney insufficiency and could maintain the stability of renal function (Bruns et al., 1991; Jindal et al., 1992; Branten et al., 1998; Branten et al., 2007; du Buf-Vereijken et al., 2004). Randomized, controlled clinical studies are still needed to confirmed the efficacy of RTX compared to glucocorticoids plus cyclophosphamide. However, compared with RTX, cyclophosphamide-based regimen was associated with much more adverse effects.

**TABLE 5:** Summary of therapeutic trials about the effectiveness and safety of cyclophosphamide in membranous nephropathy with kidney insufficiency.

*n*	Gender (M/F)	Creatinine at month 0 (µmol/L)	Follow-Up (months)	Creatinine at the End of Follow-Up (µmol/L)	Renal Function			Adverse Effects	References
					Improved	Stabilized	Deteriorated		
11	9/2	198 (159–371)	33 (12–54)	NA	7	4	0	Cushingoid changes were seen in most patients. Two patients had leukopenia. Two patients developed pneumonia	Bruns et al., 1991
9	7/2	222 (135–356)	83 (13–144)	NA	4	1	4^a^	One patient was diagnosed with carcinoma of the esophagus. Nine other complications occurred that could possibly be associated with cyclophosphamide therapy including anemia (*n* = 3), gastric erosions (*n* = 1), amenorrhea (*n* = 2), hair loss (*n* = 1), and varicella infection (*n* = 2)	Jindal et al., 1992
17	15/2	274 ± 126	26 (5–67)	174 ± 78^b^	13	3	1	Side- effects occurred in 8 patients, including respiratory tract infection (*n* = 5), leukopenia (*n* = 3), nausea (*n* = 2), anemia (*n* = 1), and malaise (*n* = 1)	Branten et al., 1998
32	26/6	159 (132–185)	23 (11–46)	123 (88–159)	13	18	1	Side effects occurred in 22 patients (69%) including leukopenia (*n* = 9), anemia (*n* = 7), infections (*n* = 14), and malaise (*n* = 3)	Branten et al., 2007
65	55/10	171 (106–512)	51 (5–132)	128 (69–1,000)	NA	NA	4	Treatment-related complications occurred in two thirds of patients, mainly consisting of bone marrow depression and infections. One patient has developed bladder cancer, and another patient had prostate cancer	du BufVereijken et al., 2004

a Three patients had significant worsening of renal function subsequent to the cessation of therapy and one developed ESRD.

b The level of serum creatinine was evaluated at 12 months. Data presented as median with range or mean ± SD, or number; ESRD, end-stage renal disease.

The main limitations of this study are the small sample size, short follow-up, and inconsistency in treatment regimens. However, our findings clearly show that individualized anti-PLA2R antibody-driven treatment strategies should be a choice for patients with membranous nephropathy and kidney insufficiency.

## 5 Conclusion

In conclusion, in the treatment of membranous nephropathy with kidney insufficiency, RTX might be an effective treatment to reduce proteinuria and maintain stable renal function. Anti-PLA2R antibodies can be used as markers to optimize individualized use of RTX.

## Data Availability

The raw data supporting the conclusions of this article will be made available by the authors, without undue reservation.
